# Awareness of Hazards of X-Ray Imaging and Perception Regarding Necessary Safety Measures to be Taken During X-Ray Imaging Procedures Among Patients in Public Sector Tertiary Hospitals of Karachi, Pakistan

**DOI:** 10.7759/cureus.4756

**Published:** 2019-05-25

**Authors:** S Tahira S Naqvi, Syeda Warda Batool, Syed Asad Hasan Rizvi, Kinaan Farhan

**Affiliations:** 1 Internal Medicine, Jinnah Medical and Dental College, Karachi, PAK; 2 Internal Medicine, Civil Hospital Karachi, Dow University of Health Sciences, Karachi, PAK; 3 Internal Medicine, Civil Hospital Karachi, Karachi, PAK

**Keywords:** harms of x-ray, safety measures, public sector hospitals, awareness, radiation hazards, x-ray imaging

## Abstract

Background

X-ray imaging is a common procedure performed on a regular basis for diagnostic purposes all over the world. The use of X-rays is increasing rapidly with the introduction of new radiation-oriented therapeutic practices. Although it carries significant diagnostic benefits, extensive exposure to X-ray imaging has been shown to be associated with multiple dose-dependent health risks. Awareness and knowledge among patients regarding the effects of X-ray imaging, therefore, becomes important. Through this study, we aimed to assess the knowledge and awareness of the hazards of X-ray imaging among different groups of patients visiting two of the public sector tertiary hospitals in Karachi, Pakistan. We also aimed to evaluate the necessary safety measures undertaken during X-ray imaging in these hospitals, and the perception of patients regarding the importance of these measures.

Materials and methods

A cross-sectional study was conducted in October and November 2018 at two well-known public sector tertiary care hospitals, Dr. Ruth KM Pfau Civil Hospital, Karachi and Jinnah Postgraduate Medical Centre, Karachi. A non-probability convenience sampling technique was adapted to recruit 200 participants for the study. A pretested questionnaire was used to assess the knowledge of radiation among patients and their perception regarding the necessary safety measures required to be undertaken during the X-ray imaging procedure. Data were entered and analyzed using the IBM Statistical Package for the Social Sciences 17.0 (IBM Corp., New York, USA). Frequencies were calculated for individual variables. Chi-square test was employed to measure the relationship between categorical variables. A p-value of <0.05 was considered to be significant.

Results

Out of 200 participants, 58% knew what radiation was, 42% did not. The relationship between the level of education of patients and the awareness of the term ‘radiation’ was found to be statistically significant (p-value = 0.003). Television was the most common source of information (65.5%). One participant (0.5%) thought that it was possible for X-ray imaging to cause cancer. Similarly, only one participant (0.5%) thought that it could cause decreased fertility, five participants (2.5%) thought it could cause burns, seven (3.5%) thought it could cause cataract, and 20 (10%) were of the view that anemia could be caused.

The majority of the participants (80.5%) thought that a lead sheet was important during the X-ray procedure for safety and protection. Most participants (71.5%) said that they were never provided with any such lead sheets. When asked if the participants requested for a lead sheet if not provided, the majority (71%) denied requesting for it. On analyzing, we found that a higher percentage of uneducated participants denied requesting a sheet compared to the educated ones. The relationship between the level of education and the choice of requesting for a lead sheet was found to be statistically significant (p-value =0.012).

Conclusions

The patients visiting the public sector tertiary care hospitals of Karachi seem to lack the knowledge and awareness regarding the hazards of ionizing radiations and the necessary safety measures required to be undertaken during X-ray imaging. More awareness programs should be conducted to increase the level of patients' awareness to protect them from unnecessary health risks.

## Introduction

Radiations are categorized as ionizing and nonionizing. Ionizing radiations, like X-rays, possess sufficient energy to separate an electron from an atom or molecule, producing free radicals in the process which are chemically unstable and highly reactive [1]. The emergence of X-ray imaging in the late 1800s has been one of the greatest discoveries in medicinal science [2]. The use of X-rays and many other ionizing radiations is increasing rapidly and extensively with the introduction of new radiation-oriented therapeutic practices [2-3].

Due to the extensive use of X-ray imaging, its effects should be fully understood. The most important factor when discussing the effects of X-rays is not the amount at a point in the air (exposure) but the amount of energy absorbed by tissue (dose) [1]. The dose-dependent adverse effects of X-rays have been linked to cancer and have been a focus for many researchers studying cancer risk in adults and children [4]. It is estimated that radiation exposure during medical imaging may be associated with 1.5% to 2% of all cancers in the United States in the future [5]. Prior to any researches on X-rays, radiologists who were exposed to significant amounts of X-rays were shown to develop severe forms of dermatoses, cataract, hematological disorders and various cancers [2]. This led to the development of a radiation safety principle known as ALARA (As Low As Reasonably Achievable) to allow the use of radiation with lowest possible doses required to achieve the desired diagnostic effect [2].

While radiations are extremely useful diagnostically, a study conducted in the UK estimated that up to 20% of medical X-rays ordered are not beneficial and only add to the unnecessary exposure in patients, contributing to 100-250 cases of cancer each year in the region [4]. Although X-ray doses for clinical purposes are relatively low, with the growing number of the population exposed to these radiations multiple times throughout life, any unnecessary imaging could possibly lead to several health-related problems in the future [4]. Awareness and knowledge among patients regarding the effects of X-ray imaging, therefore, becomes important. This awareness may help to necessitate the development of a more complete doctor-patient dialogue and effective patient participation in the clinical decision-making process [6]. By having the awareness of the effects of imaging procedure that is being conducted, the patient will tend to force the physician to explain the rationale behind his decision which will encourage a more justified use of imaging in patient evaluation (where benefits outweigh the risks). In addition, more elaborate doctor-patient interaction due to better awareness may also diminish the tendency of physicians to avoid seeking informed consent, a tendency which has been reported frequently in the literature. Surveying patients’ knowledge and experiences, and documenting their views regarding the services provided to them would, therefore, provide valuable insight which can help to improve the quality and safety of the healthcare system [7]. No study has yet been conducted in Pakistan to evaluate knowledge of X-ray imaging among the patients. We, therefore, aimed to assess the level of knowledge and awareness regarding the effects of X-ray imaging among patients visiting selected public sector tertiary care hospitals in Karachi, Pakistan. We also aimed to evaluate the necessary safety measures undertaken during X-ray imaging in these hospitals, and the perception of patients regarding the importance of these measures.

## Materials and methods

Over a period of two months of October and November 2018, we conducted a cross-sectional study at two public sector hospitals, Dr. Ruth KM Pfau Civil Hospital, and Jinnah Postgraduate Medical Centre after receiving ethical approval from research committee of the respective hospitals. We recruited 200 participants through a non-probability convenience sampling technique. The study included patients who presented to the radiology department to undergo X-ray imaging and were willing to participate in the study. We excluded patients who refused to participate, had cognitive dysfunction or neurological disease (which made them unable to understand and answer our questions), did not have the capacity to give informed consent, and/or if they were unable to understand the communication language (English or Urdu).

A questionnaire was designed comprising 15 questions to assess the experience and knowledge of patients regarding X-ray imaging, and their perception regarding the necessary safety measures required to be undertaken during the X-ray imaging procedure. A pilot study was conducted first on 10 participants before the start of the study to assess the questionnaire for any loopholes or difficulties in understanding the questions. Each questionnaire was then filled by individually interviewing each of the participants of the sample population after seeking informed consent. Participants were assured that collected data will not be misused and that every possible measure would be undertaken to preserve the confidentiality of the data.

Data were entered and analyzed using the IBM Statistical Package for the Social Sciences 17.0 (IBM Corp., New York, USA). Frequencies were calculated for individual variables and were represented as percentages in tables and figures. The chi-square test was used to measure the statistically significant relationship between the level of education and radiation knowledge as well as the choice of requesting for a lead sheet during the X-ray imaging procedure. A p-value of <0.05 was considered to be significant.

## Results

Characteristics of the study population

A total of 200 patients participated in our study. Their mean age was 35.96 ± 13.62 years with a minimum age of 12 years and a maximum of 80 years. Table 1 describes the frequency of participants according to their age group and level of education.

**Table 1:** Frequency of participants according to their age group and level of education

Characteristics	Classes	Frequency (n)	Percentage (%)
Age groups	<15 years	4	2
	15-24 years	39	19.5
	25-34 years	59	29.5
	35-44 years	50	25
	45-54 years	24	12
	55-64 years	15	7.5
	65 years and above	9	4.5
Education level	Uneducated	52	26
	Primary education (1st-5th standard)	28	14
	Secondary education (6th-9th standard)	30	15
	Matriculation	43	21.5
	Intermediate	24	12
	Bachelors	15	7.5
	Diploma	3	1.5
	Postgraduate	1	0.5
	Masters	4	2

Knowledge and awareness of X-ray imaging

All of the 200 participants had undergone an X-ray imaging before. Out of 200, 164 participants (82%) had less than three X-rays before this one, while 36 (18%) had three or more X-rays before this one.

Out of 200 participants, 116 (58%) knew what radiation was, while 84 (42%) did not. When analyzed on the basis of education, we found that the uneducated people were more likely to be ignorant about the term ‘radiation’ (59.61%) rather than being aware of it (40.38%). The relationship between the level of education and the awareness of the term ‘radiation’ was found to be statistically significant (p-value = 0.003). The television was the most common source of information (65.5%) for radiation knowledge, followed by books (25%), the internet (6.9%) and the newspaper (2.6%).

We then asked the participants about the effects of X-ray imaging. A total of 166 participants (83%) thought that it was beneficial, 18 (9%) were of the view that it was harmful, one (0.5%) thought that it was harmless, while 15 (7.5%) participants said they did not know. The most common benefit of X-ray was thought to be of that in diagnosis (78.5%). When given options about the population group to which the X-ray radiation was the most harmful (children, adults, elderly, pregnant women), the majority participants (n=182) (91%) said that they did not know, 13 participants (6.5%) correctly identified the most susceptible population group, while five (2.5%) gave an incorrect answer.

When asked about the possibility of the harms of X-ray to be increased on repeated exposure, 117 participants (58.5%) thought that it was possible, 44 thought that it was not possible (22%), 24 said that they did not know (12%), while 15 gave no answer to the question (7.5%).

The radiation knowledge of the patients was further assessed by questions presented in Table 2.

**Table 2:** Frequency of participants with their respective responses for general questions regarding radiation

Questions	Answers	n (%)
After completion of an x-ray exam, do you think that the room emits X-ray radiations?	Yes	63 (31.5%)
	No	50 (25%)
	Do not know	84 (42 %)
	No response	3 (1.5%)
In your opinion, which of the following procedure is associated with a greater dose of radiation?	CT Scan	59 (29.5%)
	Chest X-ray	20 (10%)
	Skull X-ray	36 (18%)
	Do not know	84 (42%)
	No response	1 (0.5%)
Is it necessary to adjust the dose of the radiation with respect to the age of the patient?	Yes	96 (48%)
	No	11 (5.5%)
	Do not know	84 (42%)
	No response	9 (4.5%)
Do you think that X-ray imaging can cause cancer?	Yes	1 (0.5%)
	No	184 (92%)
	No response	15 (7.5%)
Do you think that X-ray imaging can cause anemia?	Yes	20 (10%)
	No	165 (82.5%)
	No response	15 (7.5%)
Do you think that X-ray imaging can cause burns?	Yes	5 (2.5%)
	No	180 (90%)
	No response	15 (7.5%)
Do you think that X-ray imaging can cause cataract?	Yes	7 (3.5%)
	No	178 (89%)
	No response	15 (7.5%)
Do you think that X-ray imaging can cause fertility problems?	Yes	1 (0.5%)
	No	184 (92%)
	No response	15 (7.5%)

Awareness and practice of necessary safety measures during X-ray imaging

When asked if they thought a lead sheet was important during X-ray procedure for safety and protection, the majority of the participants (n= 161) thought that it was important (80.5%), 12 thought that it was not important (6%), while 27 said that they did not know (13.5%). Most participants (n= 143) (71.5%) said that they were never provided with any such lead sheets, 48 (24%) said that they were always given lead sheets during the procedure, while nine (4.5%) said that they were given lead sheets sometimes.

When asked if the participants requested for a lead sheet if not provided one, the majority of the participants denied requesting for it (n=142) (71%). Slightly more than a quarter of the participants (n= 58) (29%) said that they do request for a lead sheet.

**Figure 1:**
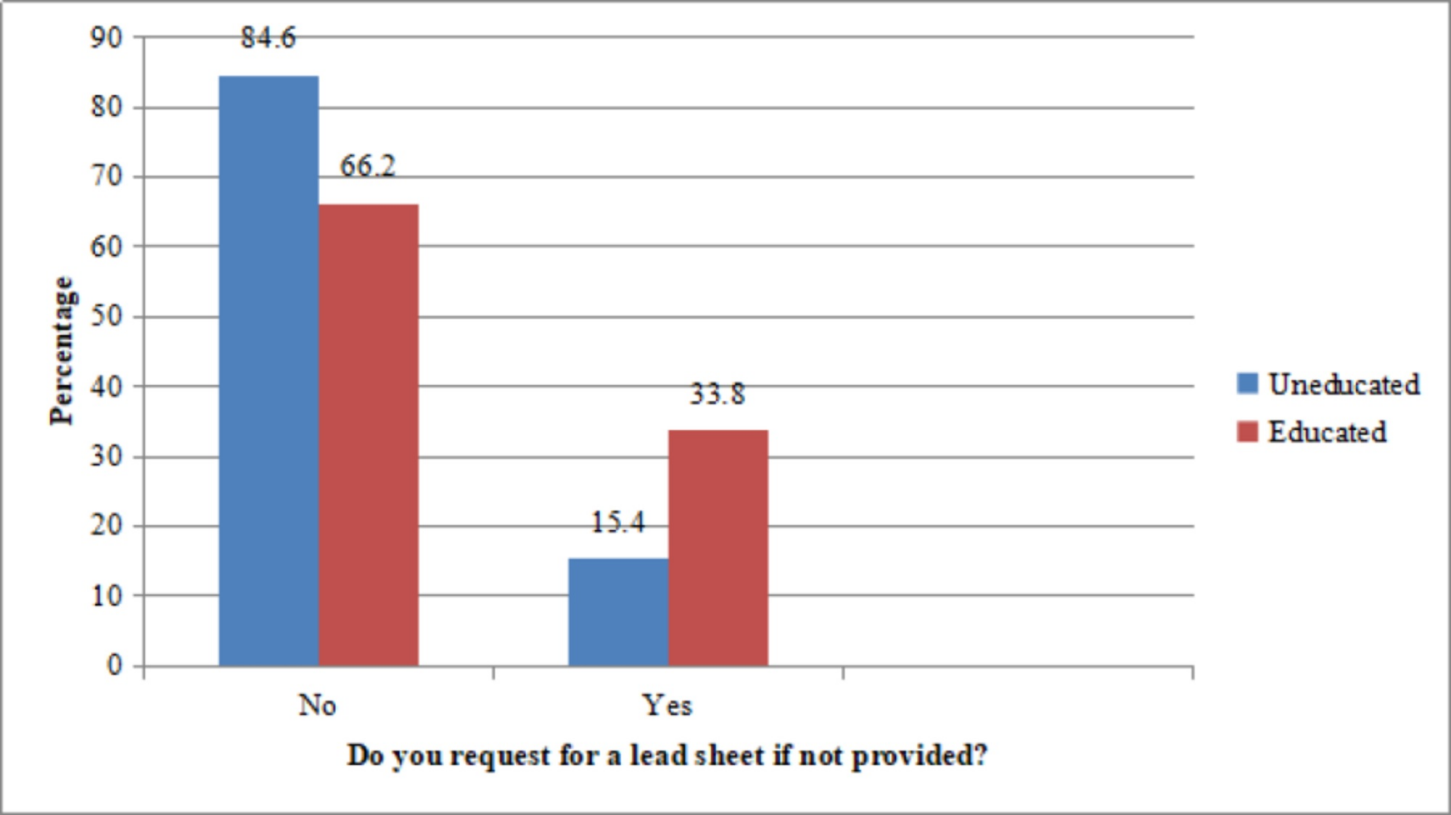
Percentage of participants with their choice of requesting for a protective lead sheet stratified by their level of education

We then analyzed this data again based on education. We found that a higher percentage of uneducated participants denied requesting a sheet as compared to the educated participants. The relationship between the level of education and the choice of requesting for a lead sheet was found to be statistically significant (p-value =0.012). Figure 1 displays the percentage of these participants with their respective responses stratified by the level of education. When asked if the lead sheet was provided upon request, 39 participants (19.5%) answered yes, 10 (5%) answered no, while 151 (75.5%) said that it was provided sometimes.

## Discussion

Through this study, we aimed to highlight the knowledge and awareness of radiation risks of X-ray imaging among the patients. Our results show that a high percentage of the study population (42%) was unaware of the term ‘radiation'. The study population demonstrated a poor level of knowledge regarding the procedure and the harmful effects of X-ray imaging altogether. These results are consistent with those that we found in the literature. A study conducted in Hong Kong reported that 87.9% of the local patients were unaware of the fact that plain X-rays contain radiations [8]. Another study reported similar results, where 34% of patients did not know that imaging may expose them to radiations [9]. As opposed to these, a study reported 70.8% of participants showing an overall understanding of the imaging technique that they were undergoing [10]. Our study also demonstrated that the local patients in Karachi were ignorant to the hazards of X-rays, showing lack of awareness to the dose-dependent risks of having cancer, anemia, burns, cataract, and fertility problems (Table 2). Similar findings have been reported in the literature. A study conducted in Nigeria reported a relatively higher percentage of patients (86.7%) who did not know about the dangers of X-ray imaging [11]. Other studies have reported underestimation of cancer risk by the patients associated with imaging [8-9, 12].

According to our results, 42% of the patients did not know that the type of imaging techniques which were associated with higher doses of radiation. Other studies have reported similar results where patients seem to underrate the equivalent dose of radiations from CT scans compared to the number of chest X-rays [8, 12]. Most of the patients in our study were unable to identify the most susceptible population group to which X-rays are most harmful. Majority of them denied having any knowledge of emission of X-ray radiations in the room after the procedure is complete (Table 2). This general lack of understanding may be attributed to the relatively higher percentage of uneducated patients in our study population (26%). The relationship between the level of education and awareness of radiation was found to be statistically significant. Similar results have been reported by Sin et al., suggesting a significant effect of education on radiation knowledge [8]. However, in contrast to our study, this particular study was performed on a highly educated population with 32.6% of participants having attended university or college.

While assessing the perception and practice of necessary safety measures during the X-ray imaging procedure, we found that 80.5% of the patients were of the view that a lead sheet was important for safety against radiations. However, the majority of the patients (71.5%) said that they were never provided with any such sheet. A study reported similar results where half of the participants were never provided with protective devices during radiation exposure [11]. Apart from this, the majority of the participants (71%) never requested a protective lead sheet. This could be explained by limited health resources and the lack of funding for tertiary care government hospitals in a developing country like Pakistan. This trend of ignoring safety precautions is however alarming, considering the frequent use of X-ray imaging in the hospitals. Uneducated patients were more likely to avoid requesting for a lead sheet, and the safety concerns of the patients were significantly associated with their level of education. These results highlight that despite understanding the importance of safety measures, uneducated individuals are less likely to demand their health rights being the less literate citizens. Educating patients regarding their basic health rights and the procedure that they would be undergoing, and seeking their informed consent, therefore, becomes absolutely vital.

Among the patients who were able to identify the risks associated with X-ray imaging, the primary source of information was television. As opposed to this, other studies report healthcare providers as being the primary source of information for the patients [9, 13]. The healthcare providers may be thought to be the most appropriate source, as they are most likely to have the complete clinical background and history of radiation exposure of each individual patient. For this reason, the study from Hong Kong reported that more than half of the participants of the study expected their doctors to explain to them about the procedure that they were undergoing [8]. Our contrasting results may be explained by the fact that healthcare providers have been frequently demonstrated to have little knowledge about radiation themselves [12-13] and thus, are poor educators. A better medical school curriculum must be devised with more focus on radiology to impart better knowledge among healthcare providers. The difference in the source of information as per our results also suggests a low trend of seeking informed consent from the patients by their doctors and briefing them about the imaging technique which is being conducted. Our results also emphasize that electronic media, being the primary source of information, may play a pivotal role in improving the overall understanding of radiation risks among the masses.

## Conclusions

The overall knowledge of the patients visiting tertiary care government hospitals of Karachi regarding radiation and its hazards is unsatisfactory. Safety protocols are less implemented in these hospitals, probably due to limited resources. To ensure the protection of patients from unnecessary repeated radiation exposure, educating patients as well as the health care providers may prove to be beneficial. Public awareness programs should be conducted on a regular basis, where electronic media could play a central role. Healthcare providers should be taught to make a justified decision of exposing their patient to radiation only when the benefit outweighs the risk. It has been suggested that patients’ exposure history must be maintained and updated after each exposure. Informed consent should be sought and a clear explanation of the imaging and its associated risks should be provided to each patient prior to the procedure.
